# 497. Fecal cytokine profiling and markers of intestinal inflammation in *Aotus nancymaae* diarrhea model for enteropathogens

**DOI:** 10.1093/ofid/ofac492.555

**Published:** 2022-12-15

**Authors:** Rhonda A Lizewski, Juan Carlos Gaspar, Roza Nuñez, Monica Nieto, Nereyda Espinoza

**Affiliations:** Naval Medical Research Unit-6, Callao, Callao, Peru; Naval Medical Research Unit No. 6, Lima, Lima, Peru; Naval Medical Research Unit 6, Lima, Lima, Peru; Naval Medical Research Unit 6, Lima, Lima, Peru; Naval Medical Research Unit 6, Lima, Lima, Peru

## Abstract

**Background:**

Naval Medical Research Unit 6 (NAMRU-6) established an animal model that closely resembles diarrheal diseases caused by *Shigella*, *Campylobacter*, and enterotoxigenic *Escherichia coli* (ETEC) in humans. These enteropathogens cause considerable mortality due to diarrhea in children and morbidity in travelers and military personnel deployed to regions of high prevalence. The successful oral challenge model developed in *Aotus nancymaae*, a New World monkey species, for *Shigella*, *Campylobacter*, and ETEC results in reproducible diarrhea attack rates of ≥70%. This model has been fundamental for the development and evaluation of new vaccine candidates. Many new approaches in vaccine development focus on stimulating antibodies against specific antigens expressed by these enteropathogens. Recent studies use the *Aotus* diarrhea model to evaluate new passive therapeutic and prophylactic treatments to prevent infection, inflammation, or diarrhea. However, the immune response and intestinal inflammation by the main enteropathogens are not clearly defined.

**Methods:**

This study investigates how the cytokines and fecal markers of intestinal inflammation respond to gastroenteritis caused by *Shigella*, *Campylobacter* and ETEC in *Aotus*. Twenty-nine fecal cytokines (FC) were measured during diarrhea induced by *Shigella flexneri* (N = 8), *Campylobacter jejuni* (N=8), and ETEC (N = 8).

**Results:**

Of note, both IL-1β and IL-15 were significantly increased at 24 hours after ETEC challenge. In contrast, when an infection with *Shigella flexneri* occurs, a significant increase in cytokines and chemokines such as MIF, MIP-1α and MIP-1β are observed 24 hours after challenge. While animals challenged with *Campylobacter jejuni* produce an increase in IL-1β 24 hours after challenge and an increase in cytokines MIF, MIP-1α and MIP-1β 4 days after challenge.

**Conclusion:**

There is nonspecific upregulation of TH1/TH17 effector cytokines and those known to mediate intestinal barrier damage. The FC profiles have a different behavior between the three enteropathogens. This provides new insights into gut-specific immune alterations in the *Aotus* diarrhea model, which will help improve the design of new prophylactic and therapeutic treatments against diarrhea.

**Disclosures:**

**All Authors**: No reported disclosures.

